# Characteristics of Corticosteroid‐Resistant Secondary Immune Thrombocytopenia Associated With Connective Tissue Diseases in China: A Retrospective Comparative Study

**DOI:** 10.1002/iid3.70236

**Published:** 2025-07-29

**Authors:** Yangchun Chen, Yingying Shi, Yuechi Sun, Yun Peng, Guixiu Shi, Yuan Liu, Shiju Chen

**Affiliations:** ^1^ Department of Rheumatology and Clinical Immunology School of Medicine, The First Affiliated Hospital of Xiamen University, Xiamen University Xiamen China; ^2^ Xiamen Municipal Clinical Research Center for Immune Diseases Xiamen China; ^3^ Xiamen Key Laboratory of Rheumatology and Clinical Immunology Xiamen China

**Keywords:** CD8^+^ T cell percentage, connective tissue disease, corticosteroid‐resistant, secondary immune thrombocytopenia

## Abstract

**Objective:**

Corticosteroid‐resistant secondary immune thrombocytopenia (ITP) is a challenging condition in clinical practice. This study aimed to explore the clinical and immunological characteristics of corticosteroid‐resistant secondary ITP associated with connective tissue diseases (CTD‐ITP).

**Methods:**

We conducted a retrospective analysis of 201 CTD‐ITP hospitalized patients between 2014 and 2022. Patients were categorized as corticosteroid‐resistant or corticosteroid‐sensitive, and their demographic, clinical, and immunological data were compared. Logistic regression analysis was employed to identify independent predictors of corticosteroid resistance.

**Results:**

Corticosteroid resistance was observed in 27.4% of patients. Compared with the corticosteroid‐sensitive group, the corticosteroid‐resistant group exhibited a higher percentage of CD3^+^ T cell (71.38% vs. 64.70%, *p* = 0.004) and CD3^+^CD8^+^ T cell (38.55% vs. 28.95%, *p* = 0.003), but a lower percentage of CD19^+^ B cell (13.70% vs. 22.45%, *p* = 0.001) in peripheral blood. No significant differences were found in other demographics, clinical features, or autoantibody profiles. The multivariable logistic regression analysis showed that higher percentage of CD3^+^CD8^+^ T cells (OR = 1.170, 95% CI: 1.014–1.350, *p* = 0.031) was an independent risk factor for corticosteroid resistance in CTD‐ITP patients.

**Conclusion:**

This study revealed the potential role of higher CD3^+^CD8^+^ T cells in corticosteroid resistance among CTD‐ITP patients, and provided the potential biomarker for predicting corticosteroid therapy response.

## 1 Introduction

Immune thrombocytopenia (ITP) is an immune‐mediated disorder characterized by impaired platelet production and platelet destruction, leading to a platelet count of < 100 × 10^9^/L and an increased risk of bleeding [1, 2]. ITP can be classified as primary and secondary ITP. Primary ITP is characterized by isolated thrombocytopenia in which no other diseases or causes linked to thrombocytopenia are found. Secondary ITP arises in association with specific predisposing factors, mainly caused by connective tissue disease (CTD), viral infections, and certain drugs. Secondary ITP associated with CTD (CTD‐ITP) is more frequent, especially with primary antiphospholipid syndrome (pAPS) or systemic lupus erythematosus (SLE) [3].

Corticosteroids (also known as steroids or glucocorticoids) are the first‐line treatment for both primary and secondary ITP [4]. However, it is reported that about 10%–30% of ITP patients are resistant to corticosteroids, which is a major challenge in ITP management [5]. Other therapies such as traditional immunosuppressants, intravenous immunoglobulins, B‐cell targeting therapies (e.g., rituximab and belimumab), and thrombopoietin‐stimulating agents are important treatments for ITP patients if they have poor response to corticosteroids [6, 7]. Identifying predictors of corticosteroid resistance is critical for optimizing treatment strategies and minimizing unnecessary side effects from prolonged steroid use. Several immune markers, including elevated regulatory T cells and Th17 levels, have been associated with steroid responsiveness in ITP [8, 9]. Nevertheless, few studies have focused on the predictors of corticosteroid resistance specifically in CTD‐ITP patients, particularly within Chinese populations.

This study aimed to bridge this gap by evaluating clinical and immunological characteristics associated with corticosteroid resistance in Chinese CTD‐ITP patients. Our findings might provide insights into the pathogenesis of corticosteroid resistance and guide personalized treatment for CTD‐ITP.

## 2 Patients and Methods

### 2.1 Study Design

**Figure 1:**
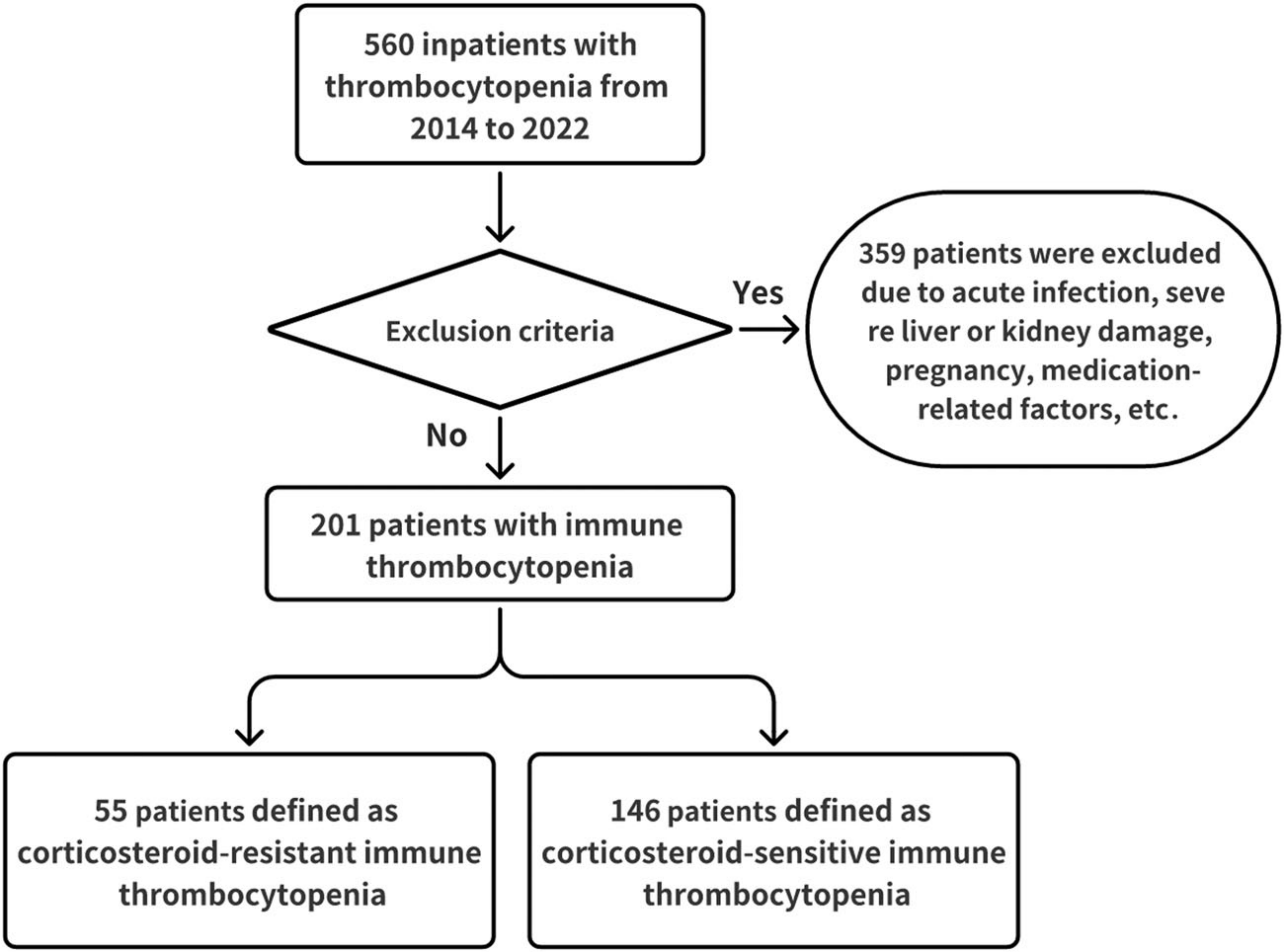
Screening process of CTD‐ITP patients.

This retrospective cross‐sectional comparative study was conducted and approved from the Clinical Research Ethics Committee at the First Affiliated Hospital of Xiamen University (XMYY‐2024KY190). All included patients were hospitalized in the Department of Rheumatology from 2014 to 2022. The only inclusion criterion was CTD‐ITP. ITP was diagnosed as a platelet count below 100 × 10^9^/L according to the International Working Group (IWG) guidelines [1]. CTDs included SLE, pAPS, primary Sjögren syndrome (pSjS), undifferentiated connective disease (UCTD), vasculitis, and rheumatoid arthritis (RA). Diagnosis of these CTDs was based on the criteria of the American College of Rheumatology (ACR) or European Alliance of Associations for Rheumatology (EULAR) [10, 11, 12, 13, 14, 15, 16, 17]. Exclusion criteria were as follows: primary ITP, other non‐CTD disorders or causes that might be associated with thrombocytopenia like drug‐induced thrombocytopenia and hematological diseases among others [18, 19], pregnant individuals, active infection (hepatitis B/C virus, or HIV), severe liver and kidney dysfunction, neoplastic diseases, and the prior use of B‐cell depleting agents. All hospitalizations attributable to ITP were evaluated for inclusion in this study. For patients with several hospitalizations, the first hospitalization was included for analysis. After removing duplicate cases, 560 unique patients were included for the initial analysis. Based on the selection criteria, 201 patients were included in the final analysis, as illustrated in the flowchart in Figure 1.

### 2.2 Corticosteroid‐Resistant ITP Definition

Corticosteroid‐sensitive ITP was defined by the absence of bleeding, with platelet counts ≥ 30 × 10^9^/L and at least a twofold increase from baseline levels within 4 weeks of corticosteroid treatment, confirmed on two separate occasions spaced more than 7 days apart [19, 20]. Corticosteroid‐resistant ITP was defined as a lack of adequate response to at least 4 weeks of standard corticosteroid treatment, typically 1 mg/kg/day of prednisone or its equivalent. This resistance was indicated by one of the following: platelet levels < 30 × 10^9^/L, platelet levels ≥ 30 × 10^9^/L but accompanied by bleeding symptoms, or failure to achieve a twofold increase from baseline levels. These criteria must be validated with two separate platelet counts taken on different days, at least 1 day apart [18, 19, 20].

### 2.3 Data Collection

Clinical data, including demographic profile (the gender, age, body mass index (BMI)), CTD‐related manifestations (rash, arthralgia, Raynaud's phenomenon, etc.), and bleeding symptoms (e.g., petechiae, purpura, gingival bleeding), were extracted from patient hospitalization records. Bleeding severity was classified according to the validated Gruppo Italiano Malattie EMatologiche dell' Adulto ITP Working Party' bleeding scale as: Grade 0 (no bleeding), Grade 1 (petechiae), Grade 2 (ecchymoses and/or dripping with moderate loss of blood), Grade 3 (major mucous hemorrhage with copious loss of blood without sequelae), or Grade 4 (major mucous and/or parenchymal hemorrhage with copious loss of blood with sequelae and/or life‐threatening or death) [20].

Lymphocyte subpopulations (CD3^+^ T cells, CD4^+^ T cells, CD8^+^ T cells, and CD19^+^ B cells) and natural killer (NK) cells (CD3^−^CD16^+^CD56^+^) were characterized using flow cytometric analysis (Molfo). Circulating blood samples were collected and stained with monoclonal antibodies targeting specific cell surface antigens, and the stained cells were then analyzed by flow cytometry to determine the percentage of each cell subset. In most cases, these measurements were taken during the first blood draw upon the patient admission. Other detailed laboratory tests and immunological tests including autoantibody profiles, complement levels, and immunoglobulin levels in the blood were collected and analyzed.

### 2.4 Statistical Analysis

Statistical analyses were conducted using SPSS Statistics 23.0 (IBM, Armonk, New York). Data normality was assessed using the Kolmogorov–Smirnov test. For continuous variables, normally distributed data were reported as mean ± standard deviation (SD) and analyzed using Student's *t*‐tests, whereas non‐normally distributed data were reported as median with interquartile range (IQR) and compared using Mann–Whitney *U* tests. Categorical data were presented as numbers with percentages. The differences between groups were evaluated using the Student's *t*‐test for normally distributed continuous variables data and the Mann–Whitney *U* test for non‐normally distributed continuous data. The Fisher exact test was applied for binary categorical data. For missing data, incomplete cases were excluded from analysis without imputation, ensuring results were based solely on available data. Binary logistic regression was conducted with corticosteroid resistance (yes/no) as the dependent variable, and variables with a significance level of < 0.2 in univariate analysis were included in the multivariable model. *p* < 0.05 was statistically significant.

## 3 Results

### 3.1 Baseline Clinical Features of Patients With Corticosteroid‐Resistant and Corticosteroid‐Sensitive CTD‐ITP

Based on the predefined eligibility criteria, 201 patients were finally included to evaluate the characteristics of CTD‐ITP (Figure 1), including 70 (34.8%) SLE, 56 (27.9%) pSjS, 42 (20.9%) UCTD, 18 (9.0%) pAPS, 4 (2.0%) systemic vasculitis, and 3 (1.5%) RA. Additionally, 8 (4.0%) patients had other CTDs, including IgG4‐related disease, autoimmune hepatitis, systemic sclerosis (SSc), and mixed connective tissue disease (MCTD). Corticosteroid resistance was found in 55/201 (27.4%) patients. The comparisons of clinical characteristics between corticosteroid‐resistant and corticosteroid‐sensitive CTD‐ITP patients were presented in Table 1. No statistically significant differences were observed in the gender, age, disease duration, and BMI (all *p* > 0.05) between the two groups. CTDs distribution was similar in two groups with most common in SLE (33.6% and 38.2%, respectively, *p* = 0.540) and pSjS (29.5% and 23.6%, respectively, *p* = 0.412).

**Table 1:** Clinical characteristics of patients with corticosteroid‐sensitive and corticosteroid‐resistant CTD‐ITP.

Characteristics	Corticosteroid sensitive (*n* = 146)	Corticosteroid resistant (*n* = 55)	*p*
Demographic characteristics			
Age of hospitalization, year	40 (29–55)	43 (35–50)	0.453
Age of diagnosis, year	40 (28–54)	42 (33–50)	0.559
Duration of CTD disease, month	1.0 (0.3–15.0)	1.0 (0.5–24.0)	0.406
Female/male (Female, %)	129/17 (88.4)	46/9 (83.6)	0.374
BMI	21.6 (19.4–24.0)	22.0 (20.3–24.0)	0.365
Associated rheumatic diseases, *n* (%)			
SLE	49 (33.6)	21 (38.2)	0.540
PSjS	43 (29.5)	13 (23.6)	0.412
UCTD	30 (20.5)	12 (21.8)	0.844
PAPS	14 (9.6)	4 (7.3)	0.814
Systemic vasculitis	3 (2.1)	1 (1.8)	1.000
RA	2 (1.4)	1 (1.8)	1.000
Other rheumatic diseases^a^	5 (3.4)	3 (5.5)	0.801
Clinical manifestations, *n* (%)			
Bleeding	84 (57.5)	35 (63.6)	0.433
Dry mouth	30 (20.5)	13 (23.6)	0.634
Arthralgia	25 (17.1)	8 (14.5)	0.660
Fever	20 (13.7)	6 (10.9)	0.599
Rash	21 (14.4)	4 (7.3)	0.173
Dry eyes	16 (11.0)	6 (10.9)	0.992
Weight loss	14 (9.6)	5 (9.1)	0.914
Raynaud's phenomenon	7 (4.8)	4 (7.3)	0.733
Oral ulcers	5 (3.4)	3 (5.5)	0.801
Photosensitization	3 (2.1)	1 (1.8)	1.000
Bleeding scores, *n* (%)			
0	62 (42.5)	20 (36.4)	0.433
1	36 (24.7)	17 (30.9)	0.370
2	48 (32.9)	18(32.7)	0.984
Basic laboratory indicators			
Leukocyte, ×10^9^/L	5.9 (4.3–8.1)	6.1 (4.1–9.0)	0.378
Neutrophils, ×10^9^/L	4.4 (2.7–7.7)	5.1 (3.1–8.1)	0.494
Lymphocytes, ×10^9^/L	1.2 (0.7–1.9)	1.1 (0.7–1.9)	0.802
Hemoglobin, g/dL	113.5 (88.0–126.3)	111.0 (94.5–134.3)	0.499
Platelet Count, ×10^9^/L	22.5 (7.0–55.5)	21.0 (7.8–37.3)	0.198
Sedimentation rate, mm/h	26.0 (11.0–51.0)	20.5 (7.0–58.3)	0.320
C‐reactive protein, mg/L	1.6 (0.8–4.1)	1.4 (0.8–5.4)	0.513
Immunosuppressant use			
Corticosteroids, *n* (%)	59 (40.4)	28 (50.9)	0.180
Hydroxychloroquine, *n* (%)	40 (27.4)	21 (38.2)	0.138
Cyclosporine, *n* (%)	11 (7.5)	9 (16.4)	0.062
Mycophenolate mofetil, *n* (%)	3 (2.1)	4 (7.3)	0.171
Immunoglobulin, *n* (%)	1 (0.7)	1 (1.8)	0.473
Tacrolimus, *n* (%)	1 (0.7)	1 (1.8)	0.473
Thalidomide, *n* (%)	1 (0.7)	2 (3.6)	0.182

*Note:* Continuous data were presented as median with 25th–75th percentiles or frequency (%).

Abbreviations: BMI, body mass index; CTD, connective tissue disease; CTD‐ITP, secondary ITP associated with CTD; PAPS, primary antiphospholipid syndrome; PSjS, primary Sjogren's syndrome; RA, rheumatoid arthritis; SLE, systemic lupus erythematosus; UCTD, undifferentiated connective disease.

a Other rheumatic diseases included IgG4‐related disease, autoimmune hepatitis, systemic sclerosis, and mixed connective tissue disease.

Bleeding was the most common clinical manifestation of CTD‐ITP (59.2%), followed by dry mouth (21.4%) and arthralgia (16.4%). However, the severe bleeding patients (bleeding scores ≥ 3) were absent. The presence of common clinical manifestations related to CTD did not differ between corticosteroid‐sensitive and corticosteroid‐resistant groups. No statistically differences were demonstrated in previous immunosuppressant use and other laboratory parameters between those two groups (all *p* > 0.05; Table 1).

### 3.2 Immunological Characteristics of Patients With Corticosteroid‐Resistant Versus Sensitive CTD‐ITP

Immunological characteristics between corticosteroid‐sensitive and corticosteroid‐resistant CTD‐ITP patients were presented in Table 2. Antinuclear antibody (ANA) positivity was found in 75.00% of CTD‐ITP patients, while extractable nuclear antigen (ENA) antibodies were present in nearly 80.21% of patients. The most common ENA autoantibody was anti‐SSA antibody (113/192, 58.85%). Platelet antibody was present in 41.94% CTD‐ITP patients. No differences of autoantibody profiles positivity, levels of immunoglobulins, complement 3, and complement 4 were revealed between patients with corticosteroid‐sensitive and corticosteroid‐resistant CTD‐ITP (*p *> 0.05) (Table 2). The percentage of T cells was elevated in corticosteroid‐resistant CTD‐ITP than corticosteroid‐sensitive CTD‐ITP (71.38% and 64.70%, respectively, *p* = 0.004), mainly the CD3^+^CD8^+^ T cell increasing significantly (38.55% and 28.95%, respectively, *p* = 0.003). However, a decreased proportion of B cells was found in the corticosteroid‐resistant group (13.7% and 22.45%, respectively, *p* = 0.001) (Table 2).

**Table 2:** Immunological characteristics of patients with corticosteroid‐sensitive and corticosteroid‐resistant CTD‐ITP.

	Corticosteroid sensitive	Corticosteroid resistant	*p*
ANA positivity, *n*/*N* (%)	109/141 (77.30)	38/55 (69.09)	0.233
Anti‐ENA antibody, *n*/*N* (%)	111/138 (80.43)	43/54 (79.63)	0.900
Anti‐SSA antibody, *n*/*N* (%)	82/138 (59.42)	31/54 (57.41)	0.799
Anti‐Ro‐52 antibody, *n*/*N* (%)	76/138 (55.07)	29/54 (53.70)	0.864
Anti‐SSB antibody, *n*/*N* (%)	24/138 (17.39)	11/54 (20.37)	0.631
Anti‐dsDNA antibody, *n*/*N* (%)	15/138 (10.87)	7/54 (12.96)	0.682
Anti‐RIB antibody, *n*/*N* (%)	20/138 (14.49)	6/54 (11.11)	0.538
Antiplatelet antibody, *n*/*N* (%)	40/84 (47.62)	12/40 (30.00)	0.063
Anti‐ACA antibody, *n*/*N* (%)	45/138 (32.61)	20/53 (37.74)	0.503
Anti‐β_2_GP1 antibody, *n*/*N* (%)	27/136 (19.85)	16/53 (30.19)	0.128
Complement 3, mg/dL	0.86 ± 0.30	0.92 ± 0.28	0.361
Complement 4, mg/dL	0.15 (0.09–0.20)	0.16 (0.10–0.21)	0.822
Immunoglobulin G, g/L	16.60 (11.60–20.55)	14.60 (11.10–18.90)	0.146
Immunoglobulin A, g/L	2.32 (1.73–3.15)	2.05 (1.50–2.92)	0.169
Immunoglobulin E, g/L	21.45 (17.55–98.75)	22.95 (9.51–324.30)	0.801
Immunoglobulin M, g/L	1.15 (0.78–1.67)	1.05 (0.66–1.67)	0.632
CD3^+^ T cell percentage	64.70 (55.30–74.50)	71.38 (64.55–85.20)	0.004*
CD3^+^CD4^+^ T cell percentage	31.92 ± 10.37	30.00 ± 10.14	0.380
CD3^+^CD8^+^ T cell percentage	28.95 (22.50–36.95)	38.55 (27.40–46.03)	0.003*
CD4^+^CD25^+^Foxp3^+^ Treg percentage	8.81 ± 2.94	7.23 ± 2.18	0.251
CD3^−^CD19^+^ B cell percentage	22.45 (12.33–37.68)	13.70 (4.10–18.60)	0.001*
CD3^−^CD16^+^CD56^+^ NK cell percentage	6.85 (3.18–12.55)	7.20 (3.35–22.70)	0.284
CD3^+^ T cell count, cell/µL	72.38 (45.99–126.90)	101.90 (60.55–172.4)	0.040*
CD3^+^CD4^+^ T cell count, cell/µL	31.86 (20.69–67.46)	39.40 (26.31–75.32)	0.414
CD3^+^CD8^+^ T cell count, cell/µL	32.61(21.94–55.52)	52.52 (28.78–87.66)	0.028*
CD3^−^CD19^+^ B cell count, cell/µL	29.28(11.50–54.07)	17.81 (5.52–43.58)	0.056
CD3^−^CD16^+^CD56^+^ NK cell count, cell/µL	8.11(2.93–14.43)	10.43 (4.61–27.26)	0.236

*Note:* Continuous data were presented as mean ± SD or median with 25th–75th percentiles.

Abbreviations: ACA, anticardiolipin antibodies; ANA, antinuclear antigen; β_2_‐GP1, β_2_‐glycoprotein 1; ENA, extractable nuclear antigen; NK cell, natural killer cell; RIB, ribosomal antibodies.

* *p* < 0.05.

### 3.3 Risk Factor for Corticosteroids Resistance in CTD‐ITP

Univariable logistic regression analysis demonstrated CD3^+^ T cells percentages (OR = 1.048, 95% CI 1.014–1.083, *p* = 0.005), CD3^+^CD8^+^ T cells percentages (OR = 1.052, 95% CI 1.015–1.090, *p* = 0.005), CD19^+^ B cells percentages (OR = 0.947, 95% CI 0.914–0.982, *p* = 0.003) were associated with corticosteroids resistance of CTD‐ITP. CD3^−^CD16^+^CD56^+^ NK cells percentages (OR = 1.046, 95% CI 0.995–1.100, *p* = 0.077), total antiplatelet antibodies (OR = 0.471, 95% CI 0.212–1.050, *p* = 0.066) were close to statistical difference in two groups (Table 3). Thus, we incorporated gender, age, CD3^+^CD8^+^ T cells percentages, CD19^+^ B cells percentages, NK cells percentages, and other relevant characters (significance level < 0.2 in univariate analysis) into the multivariable logistic regression analysis. The findings revealed that the percentage of CD3^+^CD8^+^ T cells (OR = 1.170, 95% CI: 1.014–1.350, *p* = 0.031) was independent risk factor for corticosteroid resistance (Table 3).

**Table 3:** Univariable and multivariate logistic regression analysis of corticosteroids resistance in CTD‐ITP.

Characteristic	Univariate analysis			Multivariate analysis	
	*N*	OR, 95% CI	*p*	OR, 95% CI	*p*
Gender	201	1.485, 0.619–3.562	0.376	2.990, 0.459–19.479	0.252
Age	201	1.003, 0.984–1.023	0.735	1.012, 0.952–1.076	0.698
BMI	191	1.043, 0.949–1.145	0.384	—	—
CD3^+^ T cell percentage	108	1.048, 1.014–1.083	0.005*	1.003, 0.793–1.268	0.980
CD3^+^CD4^+^ T cell percentage	106	0.982, 0.942–1.023	0.377	—	—
CD3^+^CD8^+^ T cell percentage	115	1.052, 1.015–1.090	0.005*	1.170, 1.014–1.350	0.031*
CD3^−^CD19^+^ B cell percentage	76	0.947, 0.914–0.982	0.003*	0.978, 0.808–1.183	0.816
CD3^−^CD16^+^CD56^+^ cell percentage	87	1.046, 0.995–1.100	0.077	1.136, 0.900–1.434	0.283
Immunoglobulin G	196	0.961, 0.910–1.016	0.161	0.805, 0.616–1.053	0.114
Immunoglobulin A	194	0.882, 0.693–1.122	0.306	—	—
Immunoglobulin E	40	1.000, 0.998–1.002	0.805	—	—
Immunoglobulin M	190	0.905, 0.620–1.323	0.608	—	—
Complement 3	197	1.656, 0.563–4.875	0.360	—	—
Complement 4	196	2.006, 0.050–80.734	0.712	—	—
Antiplatelet antibody	124	0.471, 0.212–1.050	0.066	2.751, 0.193–39.258	0.456
Anti‐ENA antibody	192	0.851, 0.395–1.833	0.681	—	—
ANA positivity	196	0.656, 0.328–1.314	0.235	—	—
Anti‐β_2_GP1 antibody	189	1.842, 0.877–3.871	0.107	0.251, 0.011–5.912	0.391
Anti‐ACA antibody	191	1.253, 0.648–2.423	0.503	—	—
Rash	201	0.467, 0.153–1.428	0.182	0.093, 0.002–5.439	0.252
Bleeding score	201	1.085, 0.755–1.559	0.660	—	—
CD3^+^ T cell count	107	1.001, 1.000–1.002	0.153	—	—
CD3^+^CD4^+^ T cell count	105	1.001, 0.999–1.003	0.576	—	—
CD3^+^CD8^+^ T cell count	114	1.002, 1.000–1.005	0.083	—	—
CD3^−^CD19^+^ B cell count	88	0.995, 0.987–1.003	0.236	—	—
CD3^−^CD16^+^CD56^+^ NK cell count	86	1.001, 0.998–1.004	0.558	—	—

Abbreviations: ACA, anticardiolipin antibodies; ANA, antinuclear antigen; β_2_‐GP1, β_2_‐glycoprotein 1; BMI, body mass index; ENA, extractable nuclear Antigen; NK cell, natural killer cell.

* *p* < 0.05.

## 4 Discussion

To our knowledge, the present study was the first study evaluating the clinical and immunological profiles of corticosteroid‐resistant CTD‐ITP in the Chinese population. It provided important information on the frequency of corticosteroid resistance in nearly one‐third of hospitalized CTD‐ITP patients, and hinted increased number of peripheral CD8^+^ T cells as risk factor for corticosteroid‐resistant ITP in these individuals.

Thrombocytopenia is a common complication in CTD, with the prevalence ranging from 12.03% to 34.8% in the Chinese CTD population [21, 22, 23, 24, 25]. CTD‐ITP belongs to secondary ITP with typical serological features including positive ANA and ENA autoantibodies. In the present study and our previous studies, anti‐SSA antibody was the most common ENA autoantibody in CTD‐ITP, primary ITP, and autoimmune‐featured primary ITP [21, 26]. The presence of autoantibodies reveals the autoantibody‐mediated immunopathology involved in CTD‐ITP.

Corticosteroids are the first‐line treatment for ITP treatment, with or without intravenous pulse immunoglobulin, and remain the cornerstones of CTD therapy. However, corticosteroid resistance is not rare in CTD‐ITP, which varies across different regions and populations, ranging from 10% to 60% [27, 28, 29]. In our study, corticosteroid resistance was found in 27.4% CTD‐ITP. The pathogenic mechanisms of corticosteroid‐resistant ITP are intricate and multifaceted, and there is evident heterogeneity in CTD‐ITP. Prolonged corticosteroids use increases the risk of side effects like infections, hypertension, osteoporosis, and hyperglycemia, and corticosteroid resistance in ITP further exacerbates this risk. Therefore, it is very important to identify common risk factors for corticosteroid resistance in CTD‐ITP. However, there are few reports on this aspect. Several studies were conducted to assess predictors in primary ITP. A recent external validation primary ITP cohort study in China identifies serum ferritin, and HBsAg expression as risk factors for corticosteroids resistance [5]. In pediatric ITP, proteins like fetuin B and myosin heavy chain 9 (MYH9) have shown predictive value [30]. Increased abundance of specific gut‐microbiota species, for example, phosphotransferase system and beta‐glucoside‐specific II component, was related to therapeutic responses in primary ITP [31]. Elevated basic fibroblast growth factor and trimethylamine‐*N*‐oxide contributed to corticosteroid resistance which suggested a connection to oxidative stress and inflammasome overactivation [32]. A potential involvement of long‐lived plasma cells (LLPCs) secreting antiplatelet antibodies in primary ITP patients contributed to the development of corticosteroid resistance [33]. ITP patients with anti‐GPIbα antibodies showed reduced responsiveness to corticosteroids [34]. Our results revealed the discrepancy in the peripheral of CD19^+^ B cell percentages was consistent with the autoantibody‐mediated ﻿immunopathology in CTD‐ITP, but failed to show the significant differences in total antiplatelet antibodies, antiphospholipid antibodies, autoantibody profiles, complement 3, complement 4, and four types of immunoglobulins in corticosteroid‐resistant and corticosteroid‐sensitive CTD‐ITP. One possible explanation is that autoantibody profiles might not be primary drivers of corticosteroid resistance in CTD‐ITP, where cellular immunity, particularly T cell subsets, plays a more pivotal role.

Besides humoral immunity, T cell‐mediated ﻿immunity was also reported in corticosteroid resistance. Elevated CD8^+^ T cells and decreased CD4^+^ T cells demonstrated better responses to dexamethasone [34]. Lower peripheral Th17‐cell levels were associated with reduced responsiveness to corticosteroid therapy in newly diagnosed ITP patients, suggesting Th17 cells may serve as a potential prognostic indicator and aid in stratifying therapy for corticosteroid‐resistant individuals [8]. In a recent study with 55 newly diagnosed ITP, the levels of CD8^+^CD25str^+^ Tregs were significantly higher in the corticosteroid‐sensitive group than that of the corticosteroid‐resistant group, but not the CD4^+^ Tregs [9]. In the ITP murine model, CD8^+^ T‐cells limited the severity of the thrombocytopenia and were required for an efficacious response to corticosteroid therapy [35]. Which suggested that CD8^+^ Tregs may play a key role in response to corticosteroids. More cell‐mediated cytotoxicity genes in CD3^+^ T cells in active primary ITP suggest that T‐cell‐mediated cytotoxicity serves as an alternative mechanism for cytotoxic lysis of autologous platelets in this disorder [36]. Single‐nucleotide polymorphisms in immune checkpoint genes PDCD1 and CTLA4, particularly PDCD1^+^7209 TT, CTLA4‐1577 GG, and CT60 GG genotypes, were reported to be associated with increased susceptibility to chronic ITP [37]. In this study, T cells, mainly the CD8^+^ T cells, were higher in corticosteroid‐resistant group. This emphasizes T‐cell homeostasis, as it is commonly disrupted in CTD‐ITP due to dysfunction of Tregs, leading to the loss of immune tolerance and the initiation of ITP [38, 39]. These results seemed to be contradictory to previous findings. This might be due to the more complex immune pathogenesis of CTD‐ITP different from that of primary ITP [26]. What's more, our observations of elevated cytotoxic CD8^+^ T cells in corticosteroid‐resistant secondary ITP align with Zhao and colleagues' findings that newly diagnosed ITP patients unresponsive to first‐line therapy showed significantly increased CD8^+^ T cells and reduced CD4^+^/CD8^+^ ratios [40]. These divergent findings highlight the spectrum of ITP pathophysiology: CTD‐associated secondary ITP may favor cytotoxic effector T cell dominance, while primary ITP with intact Treg activity remains GC‐sensitive. Our data thus underscore the need for subtype‐specific therapeutic strategies. Further mechanistic studies are warranted to validate these biomarkers across ITP subtypes.

There were several limitations in this study. First, the cohort size was relatively small due to the single‐center study and stringent selection criteria applied to this well‐defined CTD‐ITP subgroup. Second, the retrospective design introduced the possibility of selection bias and relied on the quality of available medical records, which might not always be complete or consistent. Third, the total antiplatelet antibodies against membrane glycoproteins were detected but did not assess specific membrane glycoprotein‐targeted autoantibodies. This may limit our ability to fully evaluate the influence of specific platelet antibodies on corticosteroid resistance. Finally, we focused on the peripheral CD8^+^ T and B cells percentage without detecting their subsets, especially some newly reported CD8^+^ T subsets in corticosteroid‐resistant CTD‐ITP. Future mechanistic studies should characterize the immune pathways driving corticosteroid resistance in CTD‐ITP, particularly focusing on CD8^+^ T subsets. Furthermore, future prospective multicenter studies with larger sample sizes, more detailed immune profiling, and complete patient data are needed to clinically validate the predictive value of our proposed CD8^+^ T‐cell signature for treatment stratification in this patient population.

In conclusion, this study showed that corticosteroid resistance was not rare in the Chinese CTD‐ITP population with 27.4%. It is critically important to identify patients highly susceptible to corticosteroid resistance prior to corticosteroid administration. Peripheral CD3^+^CD8^+^ T cell percentages were independent predictors for corticosteroid resistance of CTD‐ITP. This may provide insights into the more complex immune pathogenesis underlying corticosteroid resistance in this type of secondary ITP, beyond the humoral immunity typically associated with the condition.

## Author Contributions

**Yangchun Chen:** data curation, formal analysis, methodology, writing – original draft. **Yingying Shi:** investigation, writing – original draft. **Yuechi Sun:** data curation, investigation, methodology, project administration, validation. **Yun Peng:** data curation, formal analysis, investigation, methodology, supervision, validation. **Guixiu Shi:** project administration, resources, writing – review and editing. **Yuan Liu:** funding acquisition, resources, writing – review and editing. **Shiju Chen:** conceptualization, data curation, funding acquisition, project administration, supervision, writing – review and editing.

## Consent

The manuscript is sufficiently anonymized in line with the anonymization policy and checklist.

## Conflicts of Interest

The authors declare no conflicts of interest.

## Data Availability

The data sets generated and analyzed are available from the corresponding author upon reasonable request.
